# Hydrophobic Modification of Thermoplastic Polyurethane for Application in Waterproof and Moisture-Permeable Membranes

**DOI:** 10.3390/ma18214998

**Published:** 2025-11-01

**Authors:** Weizhu An, Ruihan Ma, Shujuan Zhang, Mingyue Wu, Chenglong Wang, Jinhuan Zheng

**Affiliations:** 1 Engineering Research Center for Eco-Dyeing and Finishing of Textiles, Ministry of Education, Zhejiang Sci-Tech University, Hangzhou 310018, China; an_weizhu@163.com (W.A.); maruihan0411@163.com (R.M.); wmy123214@163.com (M.W.); hzzjh1968@163.com (J.Z.); 2 Shengzhou Innovation Research Institute, Zhejiang Sci-Tech University, Shengzhou 312400, China; 3 Zhejiang Provincial Innovation Center of Advanced Textile Technology, Shaoxing 312000, China; shujuanz1@163.com

**Keywords:** thermoplastic polyurethane, low surface energy, breathable-waterproof textile

## Abstract

Conventional thermoplastic polyurethane (TPU) films are commonly used in the field of waterproof and moisture-permeable textiles because of their excellent mechanical properties and flexibility. However, the high water absorption of TPU films limits their application in sophisticated waterproof and moisture-permeable products, particularly in extremely humid environments, where it may compromise the waterproof performance of textiles and negatively affect the wearing comfort. Therefore, to enhance the durability of these films, TPU was hydrophobically modified with end-hydroxy polydimethylsiloxane (PDMS). Because of its unique low-surface-energy properties and excellent hydrophobicity, PDMS substantially reduces the surface energy of the films and provides them with excellent water repellency, effectively addressing the excessive water absorption issue of TPU films. On this basis, a microporous film featuring waterproof and moisture-permeable properties is produced using phase conversion technology. Compared with that of the unmodified sample, the surface energy of silicone-modified TPU (Si-TPU) decreased by 10.56 mJ/m^2^. Furthermore, the water contact angle increased from 83° to 105°, whereas the water absorption rate considerably reduced after the modification. Moreover, Si-TPU was employed for the fabrication of a microporous membrane, which displayed exceptional moisture permeability (8651.34 g/(m^2^⸱24 h)).

## 1. Introduction

Waterproof and breathable composite fabrics are highly desired in the field of outdoor sports clothing owing to their excellent performance. These fabrics effectively block external liquid water and permit the Body moisture to escape, ensuring that the wearer remains dry and comfortable [1]. The core of the fabric, which is a waterproof and breathable membrane, exhibits exceptional dual functionality. It provides excellent waterproofing and high breathability. Therefore, highly waterproof and breathable functional fabrics suitable for high-performance outdoor gear can be developed by combining the waterproof and breathable membranes with various textile materials. Currently, the most commonly used commercial waterproof and breathable membranes can be primarily categorized into two types: microporous polytetrafluoroethylene (PTFE) films, such as those by Gore-Tex, and nonporous molecular membranes based on thermoplastic polyurethane (TPU) [2]. PTFE films provide the fabrics with excellent waterproof properties and breathability owing to their microporous structure, whereas TPU films are gaining attention for their flexibility and durability. However, the increasing environmental awareness within the industry has led to a growing concern that fluorinated compounds may degrade into perfluorooctanoic acid (PFOA) or other shorter-chain fluoroalkanes, posing potential hazards to the environment and living organisms [3,4,5]. Consequently, the industry is actively pursuing the reduction in the use of PTFE films and the exploration of environmentally friendly alternatives, such as TPU films, to achieve a balance between sustainability and high performance.

However, conventional polyurethane films exhibit rapid water absorption because of their hydrophilic characteristics, decreasing their durability and considerably limiting their application in highly waterproof and moisture-permeable functional fabrics. Therefore, enhancing the hydrophobicity of TPU film surfaces while preserving their moisture permeability is crucial for improving their overall performance.

In nature, the unique phenomenon of “no water droplets sticking to” the surface of lotus leaves after heavy rain has attracted widespread attention. Research has revealed that the surface of lotus leaves is covered with rough tubercles and wax crystals with low surface energy. Small nanostructures in the middle form air cavities, causing water droplets to only be able to come into contact with a few points at the top of the rough surface of the lotus leaf, unable to wet and thus achieving the effect of hydrophobicity; similarly, water droplets cannot wet the surface because they cannot spread on the low-surface-energy wax crystals [3]. Therefore, the hydrophobic modification of the film surface can be achieved by constructing micro-nano hierarchical structures [6] or by regulating the low surface energy properties [7]. In recent years, researchers have utilized various techniques to construct rough structures on material surfaces to enhance hydrophobic properties, such as spraying nanoparticles [8,9], etching [10,11], and micro/nano structure fabrication methods like electroplating [12]. Xue et al. [13] deposited polydopamine (PDA) onto the surface of honeycomb polyurethane porous membrane (PUPM), significantly enhancing the hydrophobicity and tensile strength. Gao et al. [14] grafted modified silica particles onto the surface of PBAT/cannabis composite membranes by the immersion method. The improvement in the surface roughness of the composite membranes increased the water contact angle from 100° to 139°. However, due to the large pore diameter of the microporous membrane, its static water pressure resistance index was only 1.4 Kpa. Wang et al. [15] deposited silica nanoparticles onto PVDF micro-porous membranes and further used PFDTMS to reduce the surface energy of the film, thereby increasing the water contact angle to above 120°. However, these methods of enhancing hydrophobicity through surface deposition often have weak particle-interfacial binding strength, and for micro-porous membranes for textile applications, the stability of the hydrophobic performance is limited. Therefore, a method for structurally designing the film material inside should be considered to produce environmentally friendly film materials that combine waterproof and moisture-permeable effects.

Recently, researchers have discovered that introducing hydrophobic segments during the synthesis process not only improves stability but also achieves good hydrophobic performance [16,17,18]. Chen et al. [19] added a 3% concentration of silicone emulsion to polyurethane using the solution-gel method, and then tested the coated water-silicone-modified polyurethane coating on ancient wood. They found that under the action of hydrophobic alkyl groups, the surface water contact angle increased from 74.4° to 107.6°. Yao et al. [20] prepared a PU/SBS/PDMS nanofiber membrane using PDMS as the base material by electrospinning, with PU and SBS as the substrates. Under the action of Si-O and Si-C bonds in PDMS, the surface tension of the membrane decreased from 32.75 mN m^−1^ to 28.46 mN m^−1^, and the surface contact angle increased significantly from 119.1° to 132.2°. However, the water permeability was poor, only 4380 g/(m^2^·24 h). You et al. [21] introduced PDMS into the TPU film structure to reduce the film surface energy, and then modified the surface with a layer of silica particles to construct a composite film with uniform micro-nano hierarchical structure. The sample exhibited excellent waterproof performance and excellent self-cleaning properties with an excellent strength of 93.3 kPa. However, due to the weakening of the uniform distribution of the modified nanometer particles on the surface, the strength of the modified film decreased from 10.23 MPa to 1.39 MPa. Moreover, the problem of poor water permeability (5235 g/(m^2^⸱24 h)) also limited its application in outdoor clothing. Therefore, the challenge lies in preparing an environmentally friendly and durable TPU film with a simple process, strong hydrophobicity, certain water permeability, and good mechanical properties.

Based on the above research, this study focuses on the design of the TPU molecular structure, introducing substances with low surface energy to enhance the hydrophobicity of the polyurethane membrane surface. We used the wet phase inversion technology to prepare micro-porous membranes with adjustable honeycomb-like pores. This film has excellent moisture permeability and can be laminated with fabrics without changing the fabric style [22,23]. Meanwhile, PDMS was blended with the soft segment PTMG to control the PDMS addition ratio, thereby reducing the surface free energy of the film and improving the water-hating and non-durable issues of TPU. This TPU micro-porous membrane not only has a simple preparation process but also has excellent hydrophobicity, breathability, and mechanical properties, which can meet the potential requirements of various applications and, to a certain extent, has guiding significance for replacing PTFE membranes.

## 2. Materials and Methods

### 2.1. Materials

Polytetrahydrofuran ether glycol (PTMG, Mn = 1000), diphenylmethane diisocyanate (MDI, 98%), 1,4-butanediol (BDO, 99.0%), and titanate isopropyl ester (Ti[OCH(CH_3_)_2_]_4_, 95%) were purchased from Aladdin (Shanghai) Reagent Co. (Shanghai, China). Terminal hydroxy polydimethylsiloxane (PDMS, Mn = 2000) was purchased from Zhejiang Jiande Polymerisation New Material Co. (Jiande, China). N,N-dimethylformamide (DMF, 99.5%) was purchased from Zhejiang Tengyu New Material Technology Co. (Wenzhou, China).

### 2.2. Synthesis of Hydrophobic TPU

The reactants were dosed at an –NCO to –OH ratio of 1, and the hard segment content was 35%. The formula for calculating the hardness content is as shown in Equation (1). Diphenylmethane diisocyanate (MDI) and poly(tetramethylenefurfurylene ether glycol) (PTMG) were vacuum-dried in an oven at 80 °C, and quantitative amounts of PTMG and PDMS were added into a three-necked flask under nitrogen protection (the PTMG:PDMS molar ratios were 40:0, 40:1, 40:2, 40:3, 40:4, 40:6, and 40:8). Thereafter, a mixture of MDI was added at 40 °C, and the reaction was carried out for 30 min after the reaction by gradually increasing the temperature to 80 °C, followed by the addition of the catalyst TPU. Thereafter, the catalyst TPT (0.0012%) was added, and the mixture was reacted for 2 h. Afterward, BDO was added and rapidly stirred. The resulting material was subsequently placed in PTFE molds and cured in an oven at 100 °C for 10 h.

(1) $$\begin{array}{c} Hard\;segment\;content=\frac{m(BDO+MDI)}{mTPU} \end{array}$$

### 2.3. Preparation of TPU Films

First, the prepared TPU particles were added to an N,N-dimethylformamide (DMF) solution at a mass fraction of 25% and stirred at 28 °C until the particles were completely dissolved. Subsequently, the solution was placed on a glass plate and scraped onto release paper using a 30 μm wire rod applicator. After air drying for a certain period, the wet film was dried in an oven to obtain a nonporous TPU film.

### 2.4. Preparation of the Microporous TPU Membrane

A specific amount of silicone-modified TPU (Si-TPU) was added to a glass bottle, followed by the addition of a certain mass of DMF. The resulting mixture was stirred using a magnetic stirrer at 50 °C for 5 h and subsequently dissolved into a polyurethane solution featuring a solid content of 25%. The as-obtained mixture was ultrasonicated for 1 h for defoaming. Thereafter, it was removed and stored for future use. Afterward, the material was coated on release paper using a 30 μm wire rod and was allowed to stand for 10 min. Thereafter, the release paper was placed in a solidification bath at 25 °C (the mass fraction of DMF was 20%) for 15 min and subsequently washed with water for 5 h. Afterward, the release paper was removed and dried, obtaining a microporous polyurethane film.

### 2.5. Testing and Characterization

The membranes were tested using attenuated total reflectance–Fourier transform infrared (FTIR) spectroscopy (Nicolet 5700, Madison, WI, USA) in the range of 4000 to 500 cm^−1^. The thermal stability of the nanofibrous membranes was characterized via differential scanning calorimetry (DSC; TA Q2000, New Castle, DE, USA) at a rate of 20 °C/min within a temperature range of −80–200 °C. Furthermore, the morphological structures of the membrane were characterized using scanning electron microscopy (SEM; Sigma 300, St. Louis, MO, USA) at an applied voltage of 3 kV. The prepared TPUs were hot-pressed into films at 170 °C and 3 MPa using a hot press, and after a certain period of time, they were subjected to tensile testing based on the international standard ISO 527-3:2018 [24]. Water absorption was determined using the weighing method. Briefly, a certain amount of TPU was weighed, and its initial mass (m_0_) was measured. Thereafter, it was immersed in water in a 50 mL beaker at room temperature, and the mass (m_1_) was recorded after immersion for 2, 4, 6, 8, and 10 h. The water absorption of TPU was calculated using Equation (2).

(2) $$\begin{array}{c} \mathrm{Water}\;\mathrm{absorption}\;\mathrm{rate}/\%=\frac{m_{1}-m_{0}}{m_{0}}\times 100 \end{array}$$

The moisture permeability was determined using a moisture permeability tester (FX3150, TEXTEST, Schwerzenbach, Switzerland) in accordance with GB/T 12704.2-2009 [25]. The moisture permeability was calculated based on the changes in the mass of the moisture-permeable cups (comprising the specimen and water) within a certain period of time, as expressed in Equation (3).

(3) $$\begin{array}{c} WVT/\%=\frac{24\times ∆m}{s\times t}\times 100 \end{array}$$

Herein, WVT is the moisture permeability g/(m^2^⸱24 h); ∆m is the difference between the two measurements of the assemblage (g); s is the area of the test surface; t is the experimental time (h). The contact angle between the test liquid and the cured film was measured using a DSA20-type video contact angle tensiometer(KRÜSS, Hamburg, Germany).

The surface free energy of TPU was calculated using the HM [26] method, and the relevant parameters of the test fluid are listed in Table 1. The calculation formula is as follows:

(4) $$\begin{array}{c} \gamma_{s}=\gamma_{s}^{d}+\gamma_{s}^{p} \end{array}$$

(5) $$\begin{array}{c} 4\left(\frac{\gamma_{s}^{d}\gamma_{L}^{d}}{\gamma_{s}^{d}+\gamma_{L}^{d}}+\frac{\gamma_{s}^{P}\gamma_{L}^{P}}{\gamma_{s}^{P}+\gamma_{L}^{P}}\right)=\gamma_{L}(1+\cos\theta ) \end{array}$$

where γ^d^s denotes the calculated value of the polarity component of the solid (mJ/m^2^); γ^P^S denotes the calculated value of the dispersive force component of the solid (mJ/m^2^); and γ^d^L and γ^P^L denote the surface tension of the probe fluid (mN/m).

## 3. Results

### 3.1. Synthesis and Characterization of Modified TPUs

**Figure 1:**
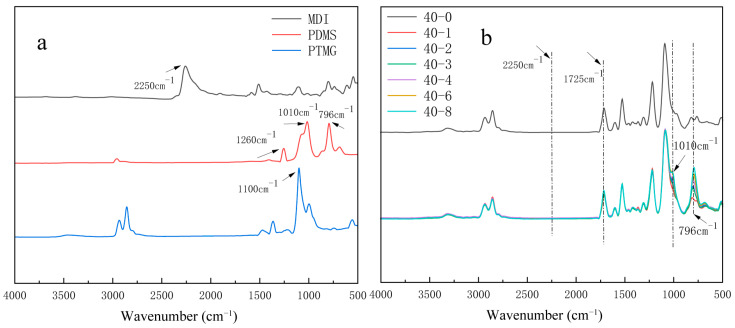
Infrared spectra of organosilicon-modified thermoplastic polyurethane (TPUs): (**a**) diphenylmethane diisocyanate (MDI), polydimethylsiloxane (PDMS), and poly(tetramethylenefurfurylene ether glycol) (PTMG); (**b**) TPUs synthesized using different PTMG:PDMS ratios.

The hydrophobic modification of TPU films by PDMS involves the reaction between hydroxyl groups and isocyanates. To confirm the success of the hydrophobic treatment, ATR-FTIR was used to characterize the raw materials (e.g., Figure 1a) and their products (e.g., Figure 1b).

The MDI spectrum depicts the characteristic absorption peak of –NCO at 2250 cm^−1^. In the end-hydroxy PDMS spectrum, the telescopic vibration absorption peak of Si–O–Si is observed at 1010 cm^−1^, and the Si–C telescopic vibration absorption peak of Si–CH_3_ is observed at 796 cm^−1^. Figure 1b depicts the complete disappearance of the characteristic peak of –NCO at 2250 cm^−1^, and the appearance of the C=O telescopic vibration peak of –NHCOO at 1725 cm^−1^, indicating the complete reaction of the isocyanate group with the carbamate group. Furthermore, compared with the 40-0 sample spectrum, the spectra of the other samples added with organosilicones exhibit the stretching vibration peaks of Si–CH_3_ and Si–O–Si at 796 and 1010 cm^−1^, respectively, indicating the successful introduction of organosilicones into the TPU linkages [27]. Additionally, the peak intensity of the Si–C bond at 796 cm^−1^ increases with increasing silicone content.

### 3.2. Effects of the PDMS Content on the Hydrophobic Properties of TPUs

**Figure 2:**
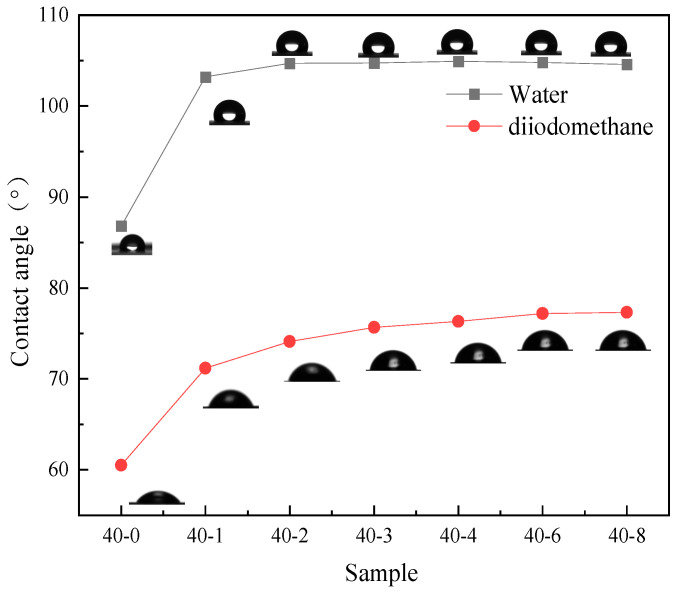
Contact angle of water and diiodomethane on the TPU films.

**Figure 3:**
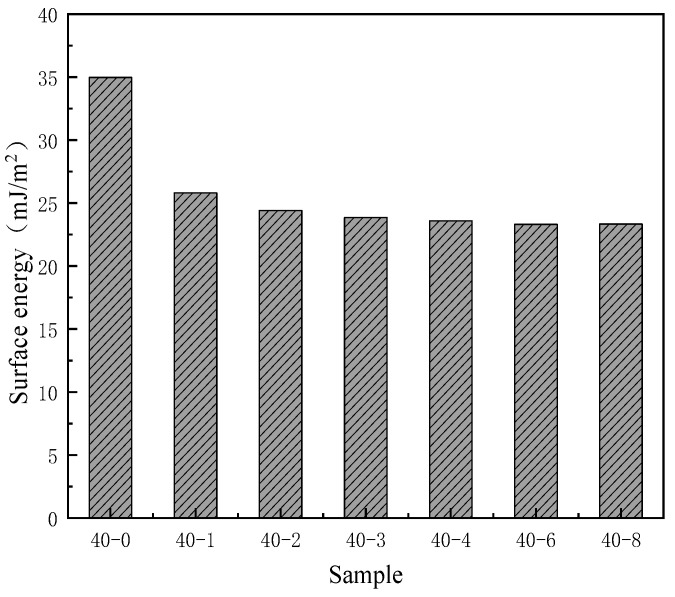
Effects of the PDMS content on the surface energy of the TPU films.

**Figure 4:**
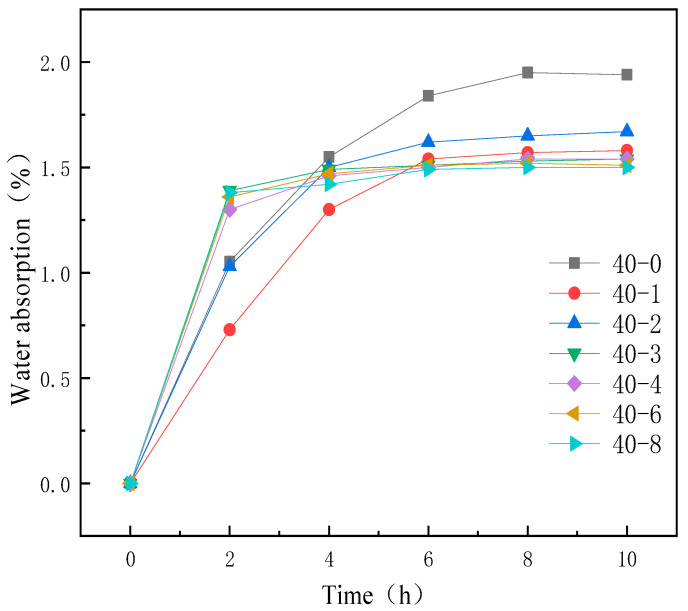
Water absorption curves of TPUs featuring different PDMS contents.

Organosilicon modification can effectively improve the hydrophobicity of TPU film surfaces. Owing to the inherently low surface energy of silicon, the introduction of its chain segments into the molecular structure of TPU can effectively improve the hydrophobicity of the TPU film surface [28]. In this context, we evaluated the hydrophobic properties of the prepared TPU films, and the results are presented in Figure 2, Figure 3 and Figure 4.

As depicted in Figure 2, with an increase in the PDMS content in the system, the water contact angle of TPU continuously increases from 83° to 105° and subsequently plateaus. This phenomenon indicates that the addition of low surface substances significantly enhances the hydrophobicity of the film. The researchers [29] also mentioned this viewpoint in the hydrophobic modification of PU materials. Similarly, the surface activity of the surface interface can be characterized by testing the surface energy to further reflect the wettability of the film surface. Since PDMS is a substance with low surface energy, its surface energy will significantly decrease after successfully introducing this component [30]. Therefore, the surface energy of the PDMS addition amount was tested, and the results showed that its surface energy also has a trend similar to the contact angle (Figure 3); with increasing silicone content, the surface energy of TPU gradually decreased and subsequently plateaued. As can be seen from Table 2, when the PTMG:PDMS ratio was 40:2, the surface energy was 24.41 mJ/m^2^, which was 10.56 mJ/m^2^ lower than that of unmodified TPU. These results are consistent with the viewpoints proposed by Lin et al. [31], but this study did not further explore the specific changes in the film’s hydrophobicity caused by the addition of low surface energy substances. To more clearly demonstrate the changes, this study further confirmed this conclusion by using the surface energy calculation formula and the changes in surface energy values before and after PDMS addition. Thus, it can be seen that PDMS can effectively reduce the surface energy of TPU, thereby enhancing its hydrophobicity. However, when the PDMS content was further increased, a corresponding improvement in hydrophobicity was not observed. This was because when the PDMS dosage exceeded a certain threshold, silica enrichment on the TPU film surface reached saturation, and the restriction of the main chain prevented the further migration of silica atoms to the surface, which affected the improvement of film hydrophobicity.

The improvement in the hydrophobicity of TPU via silicone addition was confirmed via the water absorption test, and the results are illustrated in Figure 4.

Figure 4 illustrates the water absorption curves of TPUs comprising different PDMS contents. The final water absorption of TPUs decreased with increasing PDMS content. This was because of the increased incorporation of PDMS during TPU synthesis and the migration of the hydrophobic silane chain segments of PDMS to the surface [32,33,34], which enhanced the surface hydrophobicity and prevents water from easily penetrating into the interior of the film, thus reducing the water absorption rate.

These findings further confirmed that the addition of organosilicone improved the hydrophobicity of TPU.

### 3.3. Microphase Separation

**Figure 5:**
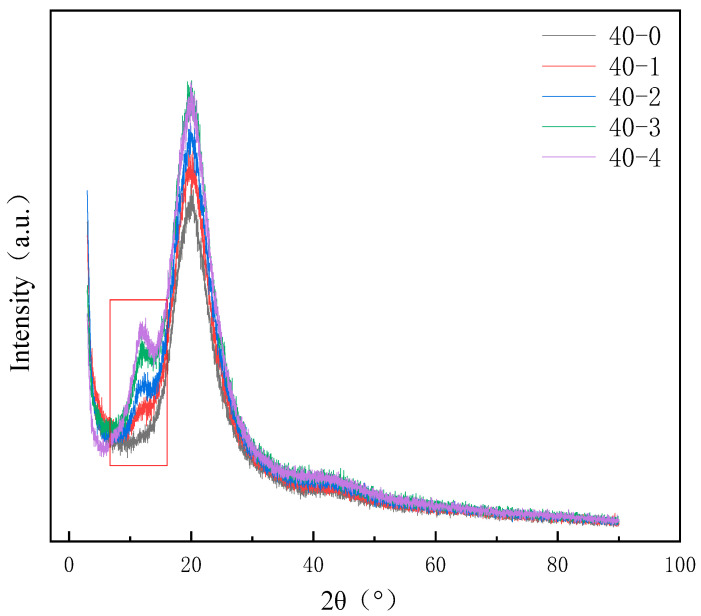
X-ray diffraction (XRD) patterns of TPUs featuring different PDMS contents.

**Figure 6:**
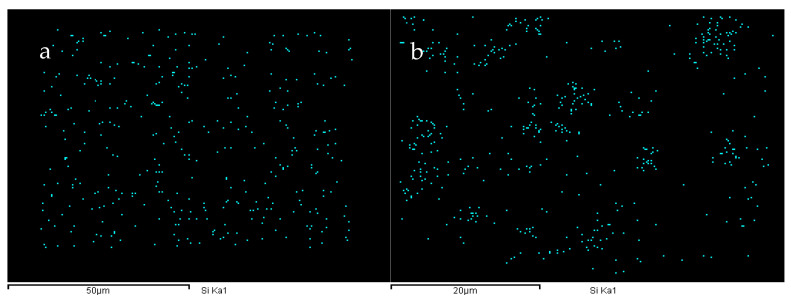
Si element distribution in the cross-sections of (**a**) 40-1 and (**b**) 40-4 TPUs.

During the synthesis of TPU, due to the differences in the dissolution parameters of the soft segment and the hard segment, there is a certain degree of microphase separation phenomenon within it. To enhance the hydrophobicity of the TPU film, we introduced PDMS with low surface energy into the system. PDMS is thermodynamically incompatible with TPU and may have an impact on the performance. To investigate the effects of PDMS on the microphase separation of TPUs, X-ray diffraction (XRD) and energy-dispersive X-ray spectroscopy (EDS) analysis results are presented in Figure 5 and Figure 6. A diffraction peak (within the frame) is observed at 2θ = 12.5°, which corresponds to the pure PDMS phase [35], and the peak intensity gradually increases with increasing PDMS content. Furthermore, the intensity of the peak between 2θ = 21° and 23° gradually increases. This is because of the incompatibility of PDMS with other phases, which increases the aggregation of the hard segment and enhances the crystallinity [16]. The addition of PDMS aids the microphase separation TPU. Moreover, the EDS energy spectra of the TPUs featuring different PDMS contents reveal that when the content of PDMS is lower, it is conducive to the increase in uniformity within the system and has a smaller impact on micro-phase separation. At this time, the distribution of Si elements in the material is more uniform (e.g., Figure 6a). When the PDMS content exceeds a certain value, it will strengthen the aggregation of the hard segment within the TPU, and the same soft segment will also aggregate to a certain extent. This situation can be confirmed by the aggregation of Si elements in the elemental analysis (e.g., Figure 6b). This phenomenon not only reflects from the side that the addition of PDMS will intensify the degree of micro-phase separation of TPU but also lays a foundation for the further study of micro-phase separation to explore the correlation between the addition amount of PDMS and the mechanical properties of TPU materials.

### 3.4. Mechanical Property Analysis

**Figure 7:**
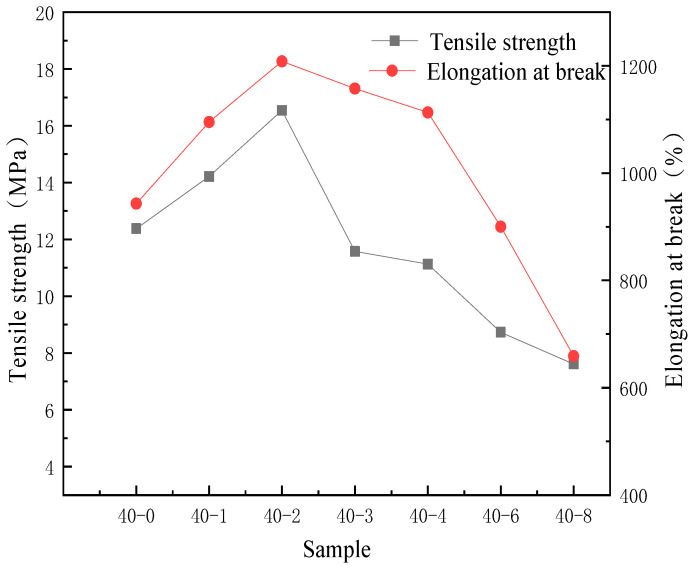
Effects of the PDMS content on the tensile strength and elongation at break of the TPU film.

As a polymer formed by the blending of soft segments and hard segments, the degree of microphase separation of TPU is closely related to its mechanical properties. Due to the poor miscibility between PDMS and TPU, the degree of microphase separation will further exacerbate the changes in the mechanical properties of the film [36]. Therefore, the correlation between the addition amount of PDMS and the mechanical properties of TPU was investigated, and the results are presented in Figure 7.

As depicted in Figure 7, with increasing PDMS content, the tensile strength and elongation at break of the samples first increase and subsequently decrease. This is because the mechanical properties of the samples are solely determined by the content of hard segments and their microstructure for specific synthetic raw materials, and the content of hard segments is 35%. Considering the microstructure, within a certain range, the incorporation of PDMS increases the disorder of the soft segments and promotes microphase mixing between hard and soft segments, enabling efficient stress transfer and thereby achieving optimal mechanical performance. Consequently, the maximum value was reached when the ratio of PTMG to PDMS was 40:2, indicating that PDMS has a certain improvement effect on the tensile properties of the film. Some studies have shown that adding PDMS to polymers can increase the degree of microphase separation [37], thereby enhancing the elasticity of the film. Based on this, through tensile testing analysis in this paper, it is concluded that a small amount of organic silicon segments can regulate the disorder of the soft segments and enhance the microphase mixing of TPU, thereby increasing the tensile properties. Concurrently, the water absorption of sample 40-2 is significantly reduced compared to unmodified TPU, exhibiting a moderate level. This improved hydrophobicity arises from the surface enrichment of silicon-containing segments, where low-surface-energy PDMS chains migrate and accumulate at the film-air interface during film formation, forming a hydrophobic surface layer. As the PDMS content is further increased, water absorption continues to decrease but eventually plateaus. This saturation occurs because the PDMS chains fully cover the material surface, forming a dense hydrophobic layer; further increases in PDMS content cannot significantly alter the surface chemical composition, and thus the hydrophobicity no longer improves markedly. However, when the PDMS content exceeds a certain critical value, compatibility within the material deteriorates. As illustrated in Figure 6b, silicon elements exhibit clear aggregation, which exacerbates microphase separation and ultimately leads to a decline in both tensile strength and elongation at break [38].

### 3.5. Effects of the PDMS Content on the Moisture Permeability of Nonporous TPU Membranes

The introduction of substances with low surface energy increases the water repellency of the TPU surface, which hinders the transmission of water vapor within the micropores and inhibits the moisture permeability. Therefore, the addition of PDMS also has a certain impact on the water vapor transmission rate.

**Figure 8:**
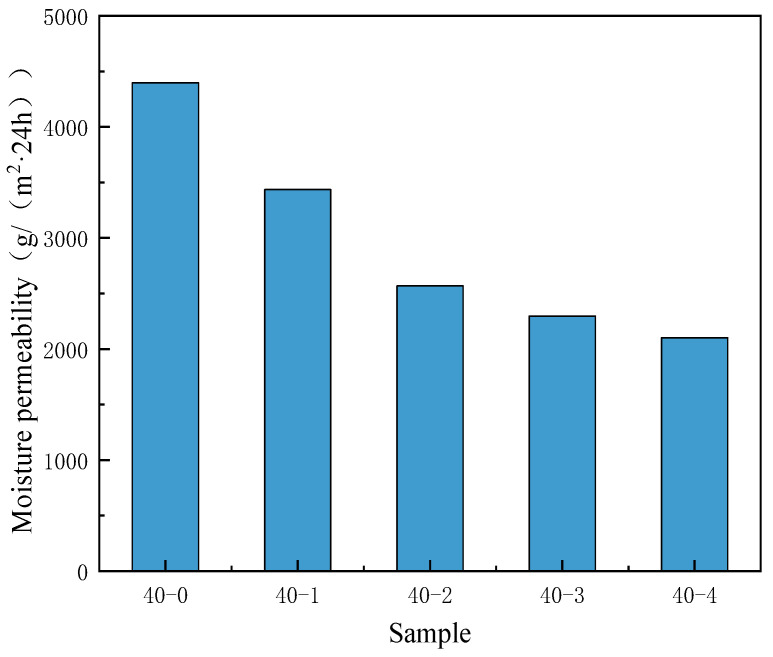
Effects of the PDMS content on the moisture permeability of nonporous TPU membranes.

As depicted in Figure 8, the moisture permeability of the nonporous TPU membrane without PDMS is 4396 g/(m^2^⸱24 h). However, when the PDMS content increased, the moisture permeability of the nonporous membrane gradually decreased. The addition of PDMS improved the hydrophobicity of the nonporous TPU membrane, whereas it decreased the moisture permeability. The research has shown that by changing the internal composition of the film, the surface energy of the film can be effectively reduced, thereby enhancing its hydrophobicity [39]. However, the enhancement of hydrophobicity inevitably leads to a loss of the film’s moisture permeability, resulting in poor moisture permeability and far below the basic requirements of waterproof and moisture-permeable films. Therefore, when improving a single property, attention should also be paid to the collaborative impact on other properties.

### 3.6. Structural and Performance Analyses of Microporous Wet Phase Conversion Membranes

The moisture permeability of films can be enhanced by constructing a microporous structure, with common preparation methods including electrospinning and wet-phase separation. However, waterproof and moisture-permeable membranes produced via electrospinning technology generally exhibit limited hydrostatic pressure resistance [40,41]. Therefore, we employed the wet-phase inversion method to fabricate microporous membranes. The fundamental principle of this method is the controlled exchange between the solvent in the polymer film and the nonsolvent in the coagulation bath. This results in phase separation, leading to the rearrangement and coalescence of polymer chains to form a microporous structure. During this process, the polymer chains rearrange and coalesce, leading to phase separation and the formation of polymer-rich and polymer-poor phases. During solidification, the polymer-rich phase constitutes the skeleton of the film, whereas the polymer-poor phase transforms into a porous structure, featuring a PTMG:PDMS ratio of 40:2 for wet phase conversion to prepare microporous films and investigated the film properties.

#### 3.6.1. Characterization of the Microporous Membrane Structure

The microscopic morphologies of the nonporous TPU membranes and microporous membranes were characterized via SEM.

**Figure 9:**
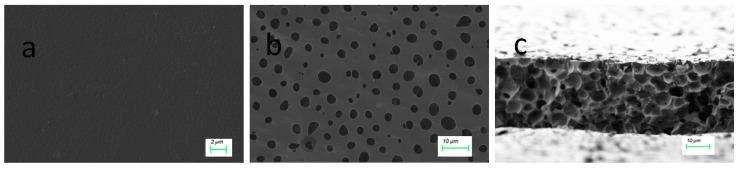
Scanning electron microscopy (SEM) images of the (**a**) nonporous TPU film and (**b**) surface and (**c**) cross-section of the microporous film, both prepared from sample 40-2.

From Figure 9, the surface of the nonporous membrane is uniformly smooth without any notable holes or cracks; however, additional micropores appear on the surface of the microporous membrane formed via wet phase conversion. The SEM image of the microporous membrane cross-section (Figure 9c) reveals that the pores in the microporous membrane prepared via wet phase conversion do not form vertical channels but assemble into a three-dimensional (3D) interconnected circular-like pore chamber structure, creating an important channel for water vapor transport in the film.

#### 3.6.2. Contact Angle Analysis

**Figure 10:**
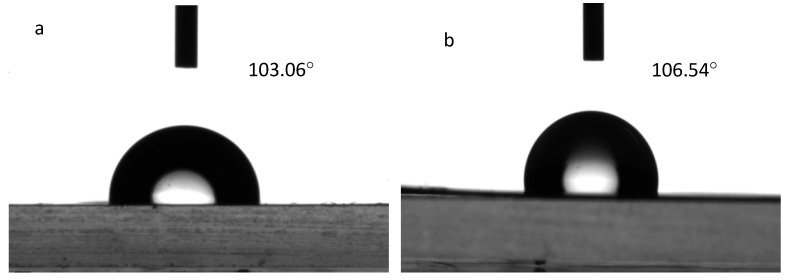
Surface contact angles of (**a**) nonporous TPU film and (**b**) microporous film.

A DSA20 video contact angle tensiometer was used to determine the surface contact angles of the nonporous and microporous films, and their surface hydrophobicity properties were compared. The results are depicted in Figure 10.

As depicted in Figure 10, the surface contact angles of the nonporous Si-TPU membrane and the microporous membrane are 103.06° and 106.54°, respectively. The contact angle of the nonporous membrane is slightly lower than that of the microporous membrane. This is because the surface wettability of a material is synergistically regulated by its chemical components and 3D surface microstructure [42]. This result is consistent with the previous research conclusions [43]. Membranes composed of Si-TPUs demonstrate a low surface free energy because of the introduction of Si–O chain segments possessing a low surface free energy [44]; consequently,, the contact angle is higher. Conversely, nonporous membranes have smooth surfaces without microscopic rough structures (Figure 9a), exhibiting relatively small contact angles [45].

#### 3.6.3. Waterproof Ability and Moisture Permeability Analyses

The waterproof ability and moisture permeability of TPU films are closely related to the wearer comfort. Therefore, we tested the moisture permeability and water pressure resistance of the films. The test results are presented in Table 3.

From Table 3, the water pressure resistance of the nonporous membrane is 31 kPa, which is higher than that of the wet phase conversion microporous membrane (19.98 kPa). This is because of the denser surface structure of the nonporous membrane without any defects, enabling higher water pressure resistance. Furthermore, with the increase in water pressure, particularly under high pressure, the microporous membrane undergoes structural deformation or even rupture, which decreases the overall water pressure resistance of the microporous membrane. The porous structure of the microporous membrane that endows it with exceptional moisture permeability. Research has shown that the presence of internal pores enhances the moisture permeability of the microporous membrane [46]. The microporous membrane prepared in this study, with its internal pores being three-dimensional interconnected, endows the film with even better moisture permeability. The water permeability rate can reach as high as 8651.34 g/(m^2^·24 h). Furthermore, This value is more than three times higher than that of nonporous membranes (2567.55 g/(m^2^·24 h)) and also exceeds the moisture transmission rate of hydrophilically modified nonporous membranes [47], highlighting the inherent advantage of constructing physical pores in achieving high moisture permeability.

## 4. Conclusions

TPU was modified with end-hydroxy PDMS for enhancing its hydrophobicity. The findings of this study revealed the substantially high comprehensive performance of Si-TPU at the PTMG:PDMS ratio of 40:2. Specifically, compared with the unmodified TPU film, the Si-TPU film prepared at the aforementioned ratio exhibited a reduction in the surface energy of 10.56 mJ/m^2^ and an increase in the contact angle from 83° to 105°, indicating its enhanced hydrophobicity. XRD and DSC analyses demonstrated that PDMS incorporation enhanced elastomeric microphase separation, improving the overall material performance. FTIR spectroscopy and SEM–EDS analyses confirmed the successful introduction of organosilicones into the polymer system, which was consistent with the desired target, confirming successful modification. Furthermore, the fabrication of Si-TPU into microporous membranes results in exceptional moisture permeability (with a moisture transmission rate of 8651.34 g/(m^2^·24 h)), successfully achieving an optimal balance between high moisture permeability and practical waterproof performance. This provides an effective technical pathway for addressing the high water absorption issues of TPU membranes while potentially serving as a substitute for PTFE membranes.

## Figures and Tables

**Table 1:** Surface tension parameters of the test liquid.

Test Fluid	γ_L_/mN·m^−1^	γ^d^_L_/mN·m^−1^	γ^P^_L_/mN·m^−1^	Polarity
Water	72.80	21.80	51.00	Polar
Diiodomethane	50.80	48.50	2.30	Nonpolar

**Table 2:** Surface energy of the silicone-modified TPU (Si-TPU) films featuring different PDMS contents.

Sample	Hard Segment Content %	PDMS Mass Fraction %	Surface Energy mJ/m^2^
40-0	35	0	34.97
40-1	35	3.09	25.81
40-2	35	5.91	24.41
40-3	35	8.48	23.85
40-4	35	10.83	23.58
40-6	35	15.00	23.32
40-8	35	18.57	23.34

**Table 3:** Moisture permeability of the Si-TPU film.

Sample	Water Pressure Resistance (kPa)	Moisture Permeability g/(m^2^·24 h)
Nonporous film	31	2567.55
Microporous membrane	19.98	8651.34

## Data Availability

The original contributions presented in this study are included in the article. Further inquiries can be directed to the corresponding author.

## References

[1] Yang G., Zhang Q., Li X., Li S., Chen H., Lu C. (2023). Preparation and property optimization of waterproof andmoisture permeable membrane made from thermally inducedfusion bonded polyurethane/polydimethylsiloxane. Fangzhi Xuebao.

[2] Qiu H., Yang Q., Cui J., Pei L., Hu G. (2023). Research progress and application of waterproof and moisture permeable membranes on textiles. Xiandai Fangzhi Jishu.

[3] Mates J.E., Ibrahim R., Vera A., Guggenheim S., Qin J., Calewarts D., Waldroup D.E., Megaridis C.M. (2016). Environmentally-safe and transparent superhydrophobic coatings. Green Chem..

[4] Cai R., Glinel K., De Smet D., Vanneste M., Mannu N., Kartheuser B., Nysten B., Jonas A.M. (2018). Environmentally friendly super-water-repellent fabrics prepared from water-based suspensions. ACS Appl. Mater. Interfaces.

[5] Zhao X., Li Y., Li B., Hu T., Yang Y., Li L., Zhang J. (2019). Environmentally benign and durable superhydrophobic coatings based on SiO_2_ nanoparticles and silanes. Colloid Interface Sci..

[6] Wang D., Ma Z., Han Z., Wu K., Liu Y., Tian X. (2025). Synthesis of high-temperature hydrophobic nanoparticles and their applications in superlyophobic coatings. J. Mater. Chem. A.

[7] Shi H., Liu W., Yang M., Liu X., Xie Y., Wang Z. (2019). Hydrophobic waterborne epoxy coating modified by low concentrations of fluorinated reactive modifier. Macromol. Res..

[8] Wang P., Zhao T., Bian R., Wang G., Liu H. (2017). Robust superhydrophobic carbon nanotube film with lotus leaf mimetic multiscale hierarchical structures. ACS Nano.

[9] Chen H., Fan H., Su N., Hong R., Lu X. (2021). Highly hydrophobic polyaniline nanoparticles for anti-corrosion epoxy coatings. Chem. Eng. J..

[10] Shen X., Gao P., Yang W., Ding Y., Bao C., Wei Z., Tian K. (2023). Study on the hydrophobic modification of PVDF membrane by low-temperature plasma etching in combination with grafting in supercritical carbon dioxide. Vacuum.

[11] Wan X., Li Y., Tian C., Zhou J., Qian S., Wang L. (2022). Fabrication and properties of super-hydrophobic microstructures on magnesium alloys by laser–chemical etching. Appl. Phys. A.

[12] Wei Z., Yan S., Lin J., Hu Q., Cui Y., Wang Q., Li Z., Cheng Z. (2024). Interiorly hydrophobic modification of electrodeposited self-supported ZnAg foam electrodes for CO_2_ electroreduction in a microchannel reactor. ACS Sustain. Chem. Eng..

[13] Xue M., Zhou D., Ji Y., Xie Y., Li C., Zhao J. (2020). The polydopamine-enhanced superadhesion and fracture strength of honeycomb polyurethane porous membranes. RSC Adv..

[14] Gao H., Sun W., Wang C., Jing M., Yang L., Gao H., Zhao R. (2023). Improving waterproof-breathable capability of degradable macroporous film/hemp hydroentangled nonwovens composite membranes by porous structure and surface wettability modification. Colloids Surf. A.

[15] Wang Q., Yang X., Zhang L.-Z. (2023). Development, testing, and molecular dynamics simulation of a membrane that resists fouling by gel pollutants for humidification–dehumidification–type seawater desalination. Desalination.

[16] Pardo-Figuerez M., López-Córdoba A., Torres-Giner S., Lagaron J.M. (2018). Superhydrophobic bio-coating made by co-continuous electrospinning and electrospraying on polyethylene terephthalate films proposed as easy emptying transparent food packaging. Coatings.

[17] Chen X., Song L., Jiang X., Zhang X. (2019). Bioinspired superhydrophobic–superhydrophilic convertible film based on anisotropic red blood cell-like particles with protuberances. Colloids Surf. A.

[18] Bernardini C., Stoyanov S., Cohen Stuart M., Arnaudov L., Leermakers F. (2011). PMMA highlights the layering transition of PDMS in langmuir films. Langmuir.

[19] Chen Y., Liu R., Xu J., Yuan X. (2023). Protection of wood in ancient timber construction by surface painting of waterborne siloxane-modified polyurethane against water destroy. Wood Mater. Sci. Eng..

[20] Yao N. (2025). Controlled Preparation and Performance Study of Fluorine-Free Nanofiber Waterproof and Breathable Membranes Modified by Organosilicon. Master’s Thesis.

[21] You X., Wang H., He J., Qi K. (2025). Fluorine-free superhydrophobic breathable membranes with lotus-leaf/corncob-like composite structure for highly water-resistant fabrics. Chem. Eng. J..

[22] Cui Y., Xu Z., Li Y., Lang X., Zong C., Cao L. (2022). Synergistic thermodynamic compatibility of polydimethylsiloxane block in thermoplastic polyurethane for flame retardant materials: Super flexible, highly flame retardant and low smoke release. Polymer.

[23] Joki-Korpela F., Pakkanen T.T. (2011). Incorporation of polydimethylsiloxane into polyurethanes and characterization of copolymers. Eur. Polym. J..

[24] (2018). Plastics—Determination of Tensile Properties—Part 3: Test Conditions for Films and Sheets.

[25] (2009). Textiles—Test Method for Water-Vapour Transmission of Fabrics—Part 2: Water Method.

[26] Bhardwaj N., Kundu S.C. (2010). Electrospinning: A fascinating fiber fabrication technique. Biotechnol. Adv..

[27] Zhang X., Li X., Zhang L. (2021). Preparation and properties of polyurethane nanofiber membrane composite fabric. Shanghai Fangzhi Keji.

[28] Fan H., Liao F., Huang S., Zhang K., Zeng D., Liu J. (2022). Exploration on the Surface Free Energy Testing Method of Paper Literatures. Zhongguo Zaozhi.

[29] Yu Y., Xu G., Zhao P., Zhang J. (2024). Biocompatible, robust, waterproof and breathable PDMS-based PU fibrous membranes for potential application in wound dressing. Mater. Today Commun..

[30] Zhangyang J., Feng D., Ma R., Zhang M., Wang C., Zheng J. (2025). Synthesis and properties of silicone-modified polyurethane acrylate for magnetically driven blue light photocurable superhydrophobic coatings. J. Text. Inst..

[31] Lin Y.-F., Wang W.-W., Chang C.-Y. (2018). Environmentally sustainable, fluorine-free and waterproof breathable PDMS/PS nanofibrous membranes for carbon dioxide capture. J. Mater. Chem. A.

[32] Calolsa R.S., Sumangala T., Kalpathy S.K., Thomas T., Kahaly M.U., Rahaman A. (2025). PDMS/Au/PDMS Multi-Layered Thin Film Composites for Hydrophobic and IR Filtering Applications. J. Appl. Polym. Sci..

[33] Chungprempree J., Charoenpongpool S., Preechawong J., Atthi N., Nithitanakul M. (2021). Simple preparation of polydimethylsiloxane and polyurethane blend film for marine antibiofouling application. Polymers.

[34] Zhao N., Xie Q., Weng L., Wang S., Zhang X., Xu J. (2005). Superhydrophobic surface from vapor-induced phase separation of copolymer micellar solution. Macromolecules.

[35] Askari F., Barikani M., Barmar M. (2013). Siloxane-based segmented poly (urethane-urea) elastomer: Synthesis and characterization. Appl. Polym. Sci..

[36] Esteghlal S., Niakosari M., Hosseini S.M.H., Mesbahi G.R., Yousefi G.H. (2016). Gelatin-hydroxypropyl methylcellulose water-in-water emulsions as a new bio-based packaging material. Int. J. Biol. Macromol..

[37] Younes G.R., Maric M. (2021). Moisture Curable Hybrid Polyhydroxyurethanes from Sugar-Derived Dicarbonates. Macromol. Mater. Eng..

[38] Yang J., Zhou S., You B., Wu L. (2006). Preparation and surface properties of silicone-modified polyester-based polyurethane coats. J. Coat. Technol. Res..

[39] Aydin A., Demirci F., Orhan M., Kocer H.B. (2019). Preparation of breathable polyurethane membranes with quaternary ammonium salt diols providing durable antibacterial property. J. Appl. Polym. Sci..

[40] Zhang Q., Liu H., Li P., Li N. (2019). Preparation and waterproof and moisture-permeable properties of electrospun polyurethane/silica composite superfine fiber membrane. J. Text. Res..

[41] Wang L., Wen Y. (2023). Preparation and Performances of Electrospun Waterproof and Wet-permeable Fabrics. J. Univ. Jinan (Sci. Technol.).

[42] Hernandez R., Weksler J., Padsalgikar A., Runt J. (2007). Microstructural organization of three-phase polydimethylsiloxane-based segmented polyurethanes. Macromolecules.

[43] Atthi N., Sripumkhai W., Pattamang P., Thongsook O., Srihapat A., Meananeatra R., Supadech J., Klunngien N., Jeamsaksiri W. (2020). Fabrication of robust PDMS micro-structure with hydrophobic and antifouling properties. Microelectron. Eng..

[44] Zhang Y., Shang J., Lv F., Chu P.K. (2012). Synthesis and characterization of novel organosilicon-modified polyurethane. Appl. Polym. Sci.

[45] Qi Z., Ziyin X., Shiqing H. (2023). Preparation and Properties of TPU/TiO_2_/PDMS CompositeFibrous Membranes. Hunan Gongcheng Xueyuan Xuebao.

[46] Anjum A.S., Son E.J., Yu J.H., Ryu I., Park M.S., Hwang C.S., Ahn J.W., Choi J.Y., Jeong S.H. (2020). Fabrication of durable hydrophobic porous polyurethane membrane via water droplet induced phase separation for protective textiles. Text. Res. J..

[47] Wang S., Sun J., Liu D., Li M. (2019). Preparation and wearability of non-porous moisture permeable TPU film. New Chem. Mater..

